# Dynamic changes and characterization of the protein and carbohydrate fractions of native grass grown in Inner Mongolia during ensiling and the aerobic stage

**DOI:** 10.5713/ajas.19.0212

**Published:** 2019-08-03

**Authors:** Zhumei Du, Na Risu, Ge Gentu, Yushan Jia, Yimin Cai

**Affiliations:** 1 Key Laboratory of Forage Cultivation, Processing and High Efficient Utilization of Ministry of Agriculture, Key Laboratory of Grassland Resources, Ministry of Education, P. R. of China, College of Grassland, Resources and Environment, Inner Mongolia Agricultural University, Hohhot 010019, China; 2 The Center of Ecology and Agrometeorology of Inner Mongolia, Hohhot 010019, China; 3 Japan International Research Center for Agricultural Sciences (JIRCAS), Tsukuba, Ibaraki 305-8686, Japan

**Keywords:** Aerobic Stability, Cornell Net Carbohydrate and Protein System, Inner Mongolian Native Grass, Silage Fermentation

## Abstract

**Objective:**

To improve the utility of native grass resources as feed in China, we investigated the dynamics of protein and carbohydrate fractions among Inner Mongolian native grasses, during ensiling and the aerobic stage, using the Cornell Net Carbohydrate and Protein System.

**Methods:**

Silages were prepared without or with lactic acid bacteria (LAB) inoculant. We analyzed the protein and carbohydrate fractions and fermentation quality of silages at 0, 5, 15, 20, 30, and 60 d of ensiling, and the stability at 0.5, 2, 5, and 10 d during the aerobic stage.

**Results:**

Inner Mongolian native grass contained 10.8% crude protein (CP) and 3.6% water-soluble carbohydrates (WSC) on a dry matter basis. During ensiling, pH and CP and WSC content decreased (p<0.05), whereas lactic acid and ammonia nitrogen (N) content increased (p<0.05). Non-protein N (PA) content increased significantly, whereas rapidly degraded true protein (PB_1_), intermediately degraded true protein (PB_2_), total carbohydrate (CHO), sugars (CA), starch (CB_1_), and degradable cell wall carbohydrate (CB_2_) content decreased during ensiling (p<0.05). At 30 d of ensiling, control and LAB-treated silages were well preserved and had lower pH (<4.2) and ammonia-N content (<0.4 g/kg of fresh matter [FM]) and higher lactic acid content (>1.0% of FM). During the aerobic stage, CP, extract ether, WSC, lactic acid, acetic acid, PB_1_, PB_2_, true protein degraded slowly (PB_3_), CHO, CA, CB_1_, and CB_2_ content decreased significantly in all silages, whereas pH, ammonia-N, PA, and bound true protein (PC) content increased significantly.

**Conclusion:**

Control and LAB-treated silages produced similar results in terms of fermentation quality, aerobic stability, and protein and carbohydrate fractions. Inner Mongolian native grass produced good silage, nutrients were preserved during ensiling and protein and carbohydrate losses largely occurred during the aerobic stage.

## INTRODUCTION

Native grasslands, including typical steppe and meadow steppe, are important grass resources in Inner Mongolia, and are distributed extensively throughout regions with a temperate semi-arid continental climate and throughout Northern Hemisphere boreal and temperate regions; they play an essential role in local animal production [1]. Inner Mongolian native grasses are rich in nutrients and optimal for livestock; they are grazed by cattle and sheep at different stages of their growth cycle [2]. Inner Mongolian native grasses are characterized by high yield and palatability, and typically meet the nutritional needs of livestock. Inner Mongolian native grasses are currently the most important feed resource on the Mongolian Plateau. Generally, Inner Mongolian native grasses are processed as hay or prepared as silage to feed animals during the winter, when food is scarce.

In China, native grassland covers 400.0 million km ^2^ and can produce 13.6 million tons of hay annually, providing approximately 42.0% of the animal feed needed in winter and early spring. However, the weather in this region is not suitable for curing hay because rainfall occurs during the harvest season, leading to leaf and nutrient loss during hay drying [3]. Therefore, methods for the long-term preservation of high-quality grass must be developed.

In Inner Mongolia, ensiling provides the principal alterna tive feed resource for local ruminants; ensiling and storing Inner Mongolian native grasses effectively covers the animal feed shortages that occur during winter on the Mongolian Plateau [4]. Silage fermentation results in good-quality feed, improving feed intake and animal production [5]. Generally, it is difficult to prepare good-quality silage from Inner Mongolian native grasses due to their low water-soluble carbohydrates (WSC) content and lactic acid bacteria (LAB) counts. Recently, a commercial LAB inoculant has been used to prepare Inner Mongolian native grass silage to increase lactic acid production, rapidly reduce pH, and inhibit the growth of harmful microbes. The use of this inoculant minimizes dry matter (DM) loss and the degradation of protein and carbohydrate, thereby preserving similar nutrient values to those of ensiled forage [6].

The Cornell Net Carbohydrate and Protein System (CNCPS) is a nutritional model that evaluates available nutritional and environmental resources within an animal production system and facilitates the formulation of diets that meet predicted animal nutritional needs. CNCPS subdivides protein into five fractions and carbohydrate into six fractions [7]. This system is more valid than simple evaluations of feed nutrient value and fermentation quality.

However, limited information is available on changes in the fermentation end-products of Inner Mongolian native grasses during silage fermentation and aerobic exposure based on CNCPS. Therefore, we investigated dynamic changes in the protein and carbohydrate fractions of Inner Mongolian native grass silage during ensiling and the aerobic stage. To improve fermentation quality, silages were prepared using a LAB inoculant. Fermentation quality and aerobic stability were also assessed.

## MATERIALS AND METHODS

### Silage preparation

The first cutting of Inner Mongolian native grass at the full bloom stage was harvested in the Xilingol typical steppe area (43° 93′ N, 116° 09′ E; Xilin Hot, Inner Mongolia, China) on 25 July 2016. The Inner Mongolian native grasses were obtained from a natural mixture of different species of grasses, including 64.32% *Stipa grandis*, 14.14% *Leymus chinensis*, 10.12% *Serratula centauroides* L. and 6.87% *Astragalus melilotoides* Pall, 2.12% *Stipa krylovii*, 1.78% *Cleistogenes squarrosa*, and 0.65% *Artemisia frigida*.

A commercial LAB inoculant, Chikuso-1 ( *Lactobacillus plantarum*, Snow Brand Seed Co., Ltd, Sapporo, Japan), was used as an additive for silage preparation. After harvest, the Inner Mongolian native grasses were immediately cut into 10 mm pieces by a chopper (130DX, ARS CO., Ltd, Osaka, Japan). A 2×5 and a 2×4 factorial arrangement was used in a completely randomized design. The silages were prepared without (control) or with LAB; the LAB were inoculated by using a sprayer (SX-MD16E-2, Shixia Holding Co., Ltd, TaiZhou, China) at 20 mg/kg as 1.0×10^5^ colony forming unit/g on a fresh matter (FM) basis, and the same amount of deionized water was sprayed on the control. All ingredients of each treatment were mixed homogeneously, and three replicates of the treated forage, which were each approximately 800 g, were packed into 1.5 L polyethylene bottle silos (Changgan, Huizhou, China). The silos were heat-sealed under vacuum conditions by using a vacuum packaging sealer (DZQ–600; Shanghai Packaging Machine Manufacturing Co., Ltd., Shanghai, China). The silages were stored in ambient temperature conditions that ranged from 20°C to 26°C, and they were opened after 5, 15, 20, 30, and 60 d of ensiling for future analysis of the fermentation end-products. Three replicated samples were used to eliminate the random error.

### Fermentation analysis

Ten grams of grass or silage samples was mixed in a blender with 90 mL of deionized water and kept in a refrigerator at 4°C for 18 h, and then filtered through filter paper (pore size 30 μm; Aoke, Taizhou, China). The fermentation products of the silage were analysed by using a cold-water extraction method as described by Cai [8]. The pH was measured with a glass electrode pH metre (PHS-3C, INESA Scientific Instrument Co., Ltd, Shanghai, China). The ammonia-N content was determined in steam distillation of the filtrates as described by Cai [8]. The organic acid content was analysed by using a high-performance liquid chromatography method as described by Cai [8]. The column was a Shodex RS Pak KC-811 (Showa Denko K.K., Kawasaki, Japan). The detector was a diode array detector, 210 nm, SPD-20A (Shimadzu Co., Ltd, Kyoto, Japan). The eluent was 3 mm HClO_4_, and its flow speed was 1.0 mL/min; the column oven temperature was 40°C.

### Protein fraction

The protein fraction was calculated by the CNCPS [7]. The feed protein fraction was divided into three fractions, including non-protein nitrogen (N) (NPN; A fraction, PA), true protein (B fraction, PB) and unavailable protein (bound true protein, C fraction, PC). The PB was partitioned into three sections (PB_l_, PB_2_, and PB_3_ fractions) based on their intrinsic rates of ruminal degradation. The PB_l_, PB_2_, and PB_3_ fractions represented the rapidly degraded protein, intermediately degraded protein and slowly degraded protein, respectively. The NPN, neutral detergent-insoluble protein (NDIP) and acid detergent-insoluble protein (ADIP) of silages were analysed according to the method of Licitra et al [9] by determining the crude protein (CP) in the neutral detergent fiber (NDF) and acid detergent fiber (ADF) residues, respectively. The soluble protein (SOLP) was measured as described by Krishnamoorthy et al [10].

The protein fractions of the ration for modelling were cal culated with the following equations according to Sniffen et al [7], and the unit for all the protein fractions except NPN is % CP; the unit for NPN is % SOLP.

The PA fraction is soluble in the buffer and consists of NPN mixtures (ammonia, peptides, and amino acids) [7] that are rapidly converted to ammonia in the rumen.

PA (%CP)=NPN (% SOLP)×0.01×SOLP (% CP)

The PB fraction is subdivided to estimate the rates of ru minal degradation. The PB1 fraction refers to the rapidly degraded protein that are soluble in borate phosphate buffer, and it is rapidly degraded protein in the rumen. It was calculated by the difference between the borate phosphate buffer insoluble protein and the NPN.

$${\text{PB}}_{1}\;(\%\;\text{CP})=\text{SOLP }(\%\;\text{CP})-\text{PA }(\%\;\text{CP})$$

The PB _2_ fraction is intermediately degraded protein, which is the insoluble protein with an intermediate degradation rate in rumen that was determined by the difference between the borate phosphate buffer insoluble N and the NDIP. A part of the PB_2_ fraction is fermented in the rumen, and others escape to the lower gut.

$${\text{PB}}_{2}\;(\%\;\text{CP})=100-\text{PA }(\%\;\text{CP})-{\text{PB}}_{1}\;(\%\;\text{CP})-{\text{PB}}_{3}\;(\%\;\text{CP})-\text{PC }(\%\;\text{CP})$$

The PB _3_ fraction is slowly degraded protein, which is insoluble in neutral detergent but soluble in acid detergent, and the value of PB_3_ was the difference between the NDIP and ADIP. It is slowly degraded in the rumen because it is associated with the cell wall [10]. A large amount of the PB_3_ fraction escapes degradation in the rumen.

$${\text{PB}}_{3}\;(\%\;\text{CP})=\text{NDIP }(\%\;\text{CP})-\text{ADIP }(\%\;\text{CP})$$

The PC fraction indicates the unavailable or bound protein, which cannot be solubilised in the acid detergent fractions. It also cannot be degraded in the rumen and intestine, and does not contribute to absorbable amino acids [10]. The PC fraction was measured by the concentration of residual N after the sample was treated with acid detergent, and expressed as percentage of the CP of sample.

PC (% CP)=ADIP (% CP)

### Carbohydrate fraction

The starch concentration was determined along with the previous extraction of soluble carbohydrate [11]. Acid detergent lignin (ADL) was determined by using the residues of ADF [12] and the ash contents to determine the ADL as ash-free residues.

The fibrous carbohydrate (FC) and non-fibrous carbo hydrate (NFC) are defined as the sequential NDF and ADF fractions of the feedstuff. The total carbohydrate (CHO) content as well as the NFC content was estimated by differences between other fractions [7]; it includes the ether extract (EE).

CHO (% DM)=100-CP (% DM)-EE (% DM)-Ash (% DM)

The carbohydrate fraction of CA, CB _1_, CB_2_, and CC were calculated according to the method of Sniffen et al [7].

The carbohydrate fraction are based on the rate of degra dation. The CA fraction is rapidly fermented in the rumen by bacteria and is primarily composed of sugars in the water–soluble pool; it is also composed of organic acids and short oligosaccharides.

$$\text{CA }(\%\;\text{CHO})=[100-\text{Starch }(\%\;\text{NFC})]\times [100-{\text{CB}}_{2}\;(\%\;\text{CHO})-\text{CC }(\%\;\text{CHO})]/100$$

The CB _1_ fraction indicates the soluble fiber, starch and pectin, and it has a slower rate of digestion than the CA fraction, ranging from 0.05 to 0.50/h.

$${\text{CB}}_{1}\;(\%\;\text{CHO})=\text{Starch }(\%\;\text{NFC})\times [100-{\text{CB}}_{2}\;(\%\;\text{CHO})-\text{CC }(\%\;\text{CHO})]/100$$

The CA and CB _1_ fraction equations do not directly incorporate NDF. Starch was calculated in terms of NFC.

$$\text{NFC }(\%\;\text{CHO})=100-{\text{CB}}_{2}\;(\%\;\text{CHO})-\text{CC }(\%\;\text{CHO})$$

The main components of the CB _2_ fraction in the carbohydrate fraction are degradable cell wall carbohydrate, which have a slow rate of digestion.

$$\begin{array}{l} {\text{CB}}_{2}\;(\%\;\text{CHO})=100\times [\text{NDF }(\%\;\text{DM})-\text{NDIP }(\%\;\text{CP})\times 0.01\times \text{CP }(\%\;\text{DM})\\ -\text{NDF }(\%\;\text{DM})\times 0.01\times \text{ADL }(\%\;\text{NDF})\times 2.4]/\text{CHO }(\%\;\text{DM}) \end{array}$$

The CC fraction represents unavailable cell wall carbo hydrate, and it mainly contains lignin. In the CB_2_ and CC equations of CNCPS, the undegradable pool is calculated using lignin as a ratio to NDF [13]. This ratio is then multiplied by a factor of 2.4.

CC (% CHO)=100×[NDF (% DM)×0.01×Lignin (% NDF)×2.4]/CHO (% DM)

### Aerobic stability analysis

For the aerobic stability test, the Inner Mongolian native grass silages in each silo were opened at 60 d after ensiling. Then, approximately 3.5 kg of each replicate was pooled, mixed thoroughly, loosely packed into polyethylene jars (5 L capacity, Changgan Co., Ltd, China) and kept at room temperature (20°C to 26°C; average 23.4°C). After 0.5, 2, 5, and 10 d of aerobic exposure, the fermentation end-products, the protein and carbohydrate fractions and aerobic stability were analysed.

### Chemical analysis

The DM contents of the Inner Mongolian native grass and silages during ensiling or the aerobic stage were measured after sample drying in a forced-air oven (TY – ZK - 4, Tong Yun Co., Ltd, Suzhou, China) at 68°C for 48 h [14]. Then, the samples were ground to pass through a 1.0 mm screen (FW 100, Taisite Instrument Co., Ltd., Tianjin, China) for chemical analysis. The ash, CP and EE contents were determined according to methods 942.05, 976.05, and 920.39, respectively of Association of Official Analytical Chemists (AOAC) [14]. The organic matter (OM) content was calculated as the weight loss upon ashing. The contents of NDF and ADF were determined according to the method of Van Soest et al [12] by using an ANKOM A200i fiber analyser (ANKOM Technology, Macedon, NY, USA). The WSC content was determined by an enzymatic method [15].

### Statistical analysis

The chemical composition, fermentation quality, protein and carbohydrate fractions of the silage at 5, 15, 20, 30, and 60 d of ensiling or at 0.5, 2, 5, and 10 d of aerobic stage were analysed in a completely randomized design with 2×5 (additives [A]×ensiling d [D]) and 2×4 (additives [A]×stage d [D]) factorial treatment structures, respectively. The two-way analysis of variance procedure of SAS version 9.2 (SAS Institute Inc, Cary, NC, USA) was used for the composition analysis, and the statistical model as follows:

$${\text{Y}}_{\text{ijk}}=\mu +\alpha_{\text{i}}+\beta_{\text{j}}+\alpha \beta_{\text{ij}}+{ɛ}_{\text{ijk}}$$

where Y_ijk_ = observation, μ = general mean, α_i_ = additive effect (i = control and LAB), β_j_ = ensiling day treatments effect or stage day treatment effect (j = 1 to 5 and 1 to 4, respectively), αβ_ij_ = additive×ensiling day treatment effect or additive×stage day treatment effect, and ɛ_ijk_ = residual error.

Duncan’s multiple range comparison test was used. The differences among mean values were regarded as significant at p<0.05 [16].

## RESULTS

The chemical composition of the Inner Mongolian native grass silage during ensiling is shown in Table 1. The Inner Mongolian native grass contained 93.4% OM, 10.8% CP, 1.2% EE, 64.6% NDF, 35.9% ADF, and 3.6% WSC on a DM basis. During ensiling, the OM, EE, NDF, and ADF contents did not markedly change, and they were 93.3% to 93.4%, 1.16% to 1.23%, 64.4% to 64.6%, and 35.8% to 35.9% of DM, respectively. However, the CP and WSC contents decreased during ensiling (p<0.05). At 60 d of ensiling, the chemical composition of the control and LAB-treated silages did not markedly differ. The chemical composition was not influenced by the additive (A). The CP and WSC contents were influenced (p< 0.001) by ensiling d (D) and the interaction (A×D), whereas the OM, EE, NDF, and ADF contents were not.

The fermentation quality of the Inner Mongolian native grass silage during ensiling is shown in Table 2. The DM contents of the control and LAB-treated silages were similar, at 53.76% to 53.82%. During ensiling, the lactic acid, acetic acid and ammonia-N contents increased (p<0.05), and the pH decreased (p<0.05); the propionic acid and butyric acid contents in both silages were below the detectable level (<0.01% of FM). After 30 d of ensiling, the pH values in all silages were reduced to 4.18–4.20, while their lactic and acetic acid contents exceeded 1.0% and 0.4% of FM, respectively. The pH and lactic acid, acetic acid and ammonia-N contents of the control and LAB-treated silages did not greatly differ. The fermentation products in all silages were not influenced by A or their interaction. The pH and lactic acid, acetic acid and ammonia-N contents were influenced (p<0.0001) by D, but the DM was not influenced by D.

The protein fractions of the Inner Mongolian native grass silage during ensiling are shown in Table 3. Before ensiling, the PA, PB_1_, PB_2_, PB_3_, and PC contents of the Inner Mongolian native grass were 35.6%, 9.3%, 42.4%, 10.8%, and 2.8% based on a CP, respectively. At 5 d of ensiling, PA increased (p<0.05), the PB_1_ and PB_2_ fractions decreased (p<0.05), and PB_3_ and PC did not change. After 5 d of ensiling, the PA, PB_1_, and PB_2_ fractions remained stable. The protein fractions did not greatly differ between the control and LAB-treated silages. The protein fraction was not influenced by A and A×D. D (p< 0.001) influenced the PA, PB_1_, and PB_2_ contents but not the PB_3_ and PC contents.

The carbohydrate fraction of the Inner Mongolian native grass silage during ensiling is shown in Table 4. Before ensiling, the CHO and NFC contents were 84.3% of DM and 31.2% of CHO, respectively. The CA, CB_1_, CB_2_, and CC contents were 12.9%, 2.3%, 64.6%, and 4.5% of CHO, respectively. During ensiling, the CHO, CA, CB_1_, and CB_2_ contents decreased (p< 0.05), while the other carbohydrate fraction did not change. The carbohydrate fraction were no markedly different between the control and LAB-treated silage, and they did not influenced by A and A×D. D (p<0.05) influenced the CHO, CA, CB_1_, and CB_2_ contents but not CC and NFC.

The chemical composition of the Inner Mongolian native grass silage during the aerobic stage is shown in Table 5. The chemical composition of the LAB-treated silage did not differ from that of the control. During the aerobic stage, the OM, CP, EE, NDF, and WSC contents (p<0.05) decreased, while the ADF content increased in both silages during aerobic exposure. A had no effect on the chemical composition during the aerobic stage. D influenced (p<0.0001) the OM, CP, EE, NDF, ADF, and WSC contents, and A×D did not influence the chemical composition of either silage.

The changes in the fermentation quality of the Inner Mon golian native grass silage during the aerobic stage are shown in Table 6. During the aerobic stage, the DM, pH, and ammonia-N content increased (p<0.05), while the lactic and acetic acid contents decreased (p<0.05) in both silages. The DM and fermentation products did not markedly differ between the control and LAB-treated silages. A and A×D had no influence on the DM and fermentation products, whereas D had a influence (p<0.0001).

The changes in the protein fractions of the Inner Mongo lian native grass silage during the aerobic stage are shown in Table 7. During the aerobic stage, the PB_1_, PB_2_, and PB_3_ contents (p<0.05) decreased, and the PA and PC contents (p<0.05) increased. The protein fractions were similar in the control and LAB-treated silages. D influenced (p<0.0001) the protein fractions, while A and A×D had no effect on the protein fractions.

The carbohydrate fraction of the Inner Mongolian native grass silage during the aerobic stage are shown in Table 8. During the aerobic stage, the CHO, CA, CB_1_, and CB_2_ contents (p<0.05) decreased in both silages, while the CC and NFC contents did not change. A and A×D did not influence the carbohydrate fraction or NFC, while D influenced the CHO, CA, CB_1_, and CB_2_ contents (p<0.05) but not the CC and NFC contents.

## DISCUSSION

### Chemical composition of Inner Mongolian native grass silage

On the Mongolian Plateau, native grassland is commonly used for grazing cattle and sheep and other activities related to livestock production [1]. Traditional livestock production on the Mongolian Plateau involves grazing in summer and hay feeding in winter [17]. The major constraint on livestock production is the shortage of high-quality feed in winter. When livestock are fed high-quality roughage, production increases [18]. In summer, animals grazing on Inner Mongolian native grasses gain weight due to the superior nutrient content and palatability of these grasses compared to other forages. In this study, Inner Mongolian native grasses had relatively high CP content, exceeding 10% of DM. The dominant species in the Inner Mongolian native grassland were *Leymus chinensis* Trin., *Serratula centauroides* L., and *Astragalus melilotoides* Pall., all of which have high CP content.

Generally, LAB, aerobic bacteria, coliform bacteria, molds, and yeasts are present in Inner Mongolian native grasses before ensiling; aerobic bacteria are the dominant microorganisms during the first stage of ensiling, causing a degree of fermentation loss [6]. In our study, WSC and CP content decreased during ensiling. Some microorganisms involved in silage fermentation degrade protein and increase ammonia-N content in silage. During silage fermentation, epiphytic LAB usually become dominant and use WSC to produce lactic acid, reducing pH and inhibiting the growth of harmful bacteria [8]. Although LAB convert most WSC into lactic acid, the proliferation of other microbes can result in CP loss.

### Fermentation quality of Inner Mongolian native grass silage

Native grasses do not grow on the Inner Mongolian Plateau during winter due to the extreme cold; the resulting feed shortages restrict livestock production. Methods for preparing and storing silage from Inner Mongolian native grasses have been developed to address this problem, to produce silage with high nutritive value for increased animal production [19]. Silage is the most effective type of feed available in winter. Due to summer rains, Inner Mongolian native grasses are usually harvested in autumn. In this study, the DM content of fresh Inner Mongolian native grasses exceeded 53.5%, higher than those of other forage crops or grasses. The generally dry climate and delayed harvest in this region may result in high DM content among native grasses. Generally, farm silage is based on natural lactic acid fermentation; epiphytic LAB from forage transforms WSC into organic acids during the ensiling process. Consequently, pH is reduced and forage is preserved [20].

The LAB are normally present in Inner Mongolian native grasses along with aerobic bacteria, coliform bacteria, molds, and yeasts. Aerobic bacteria are dominant before ensiling; however, LAB are dominant during fermentation. Some epiphytic LAB grow well at low pH and produce more lactic acid in silage environments, altering microorganismal communities and influencing silage fermentation [21]. As shown in Table 2, lactic acid content exceeded 1.0% of FM, pH was lower than 4.2, and ammonia-N content was below 0.5% in all Inner Mongolian native grass silages. The fermentation process was similar between the control and LAB-inoculated silages, perhaps because epiphytic LAB produced more lactic acid, such that it approached the level produced by the LAB inoculant. Natural silage fermentation may have beneficial effects, including promotion of lactic acid production, inhibition of *Clostridia* growth (which breaks down protein), and decreased ammonia-N content. When Inner Mongolian native grasses contain sufficient natural LAB, it is unnecessary to use a LAB inoculant to prepare silage.

### Changes in protein fractions during ensiling

Protein is typically partitioned into three fractions: PA, PB, and PC [22]. PA is a direct indicator of protein hydrolysis; peptides and free amino acids are hydrolytic products that can be used as substrates for deamination and are more precise indicators of ruminant production than other protein fractions. PB is a potentially degradable true protein [22], which is further fractionated into three subfractions based on their inherent rates of ruminal degradation: PB_l_ is rapidly degraded in the rumen, PB_3_ is degraded slowly, and PB_2_ is degraded at an intermediate rate.

Wang et al [23] reported that silage inoculated with LAB had a smaller proportion of PA than the control. In our study, there was no significant difference in PA between the control and LAB treatments; both silages had low PA content. One possible reason for this result is that Inner Mongolian native grasses ferment relatively well, at a level similar to that of LAB-inoculated silage. The dominance of epiphytic LAB is preferable, as rapid acidification inhibits the growth of undesirable microorganisms and reduces protein degradation during ensiling. Li et al [24] reported that PA and PB_2_ are the main CP fractions in alfalfa silage, comprising 46.2% and 36.5% of the CP, respectively. PA and PB_2_ were also dominant in our study, comprising 35.6% and 42.4% of CP, respectively. PA, including ammonia, peptides, and amino acids, is rapidly converted into ammonia in the rumen. Nearly all soluble protein in silage and cut forage is in the form of non-protein N [22]. The difference between buffer-insoluble protein and protein that is insoluble in neutral detergent is used to estimate the PB_2_ fraction. Some of the PB_2_ fraction is fermented in the rumen, and some passes to the lower gut. The rate of digestion of the PB_2_ fraction depends on the relative rates of digestion and passage, which are affected by glutelin protein, which is found in small grains. When forage is ensiled, extensive protein hydrolysis occurs, resulting in increased soluble PA and decreased PB_1_ and PB_2_.

As shown in Table 3, during the first 5 days of ensiling, PA increased rapidly, whereas PB_1_ and PB_2_ decreased. Subsequently, levels did not change significantly until 60 d of ensiling. This pattern was caused by degradation of CP to PA, occurring mainly during the first stage of ensiling, whereas the degradation of peptides and free amino acids occurred throughout the ensiling process [24]. When forage is harvested, plant proteases dominate the true protein degradation process in the first phase of hydrolysis, whereas microbial enzymes play a primary role in the conversion of free amino acids into ammonia in the subsequent deamination phase. Therefore, PA content increased and PB_1_ and PB_2_ content decreased during ensiling.

PC is the unavailable or bound protein that comprises the ADIP fraction, which contains proteins associated with lignin, tannin-protein complexes, and Maillard products, which are highly resistant to microbial and mammalian enzymes [10]. Li et al [24] reported that PB_3_ and PC increased due to the Maillard reaction caused by heat accumulation after ensiling. In our study, however, the PB_3_ and PC content of Inner Mongolian native grass silages did not differ significantly. A possible explanation for this result is that PB_3_ and PC were slowly degraded true protein and insoluble CP, respectively, and were therefore difficult for plant proteases or microbial enzymes to degrade.

### Changes in carbohydrate fraction during ensiling

The carbohydrate fraction is the largest component of ration for cows; it can predict dietary energy and protein supply for dairy cows based on feed chemistry and rates of digestion and passage [25]. The carbohydrate fraction can also be partitioned into FC and NFC. The FC is further subdivided into CB_2_ and CC. The feed carbohydrate fraction scheme subdivides NFC into two aggregated fractions: CA, which includes organic acids and sugars, and CB_1_, which includes starches and pectin [7]. The carbohydrate fraction occur in the following decreasing order: soluble fiber, lactic acid, sugar, NDF, starch, and NFC [25]. The CA fraction is water-soluble and fermented very rapidly; it is largely composed of sugars, but also contains organic acids and short oligosaccharides [25]. Organic acids are not used as efficiently as sugars for microbial growth. In our study, the CA and CB_1_ content of the carbohydrate fraction decreased during ensiling. The LAB and yeasts that are involved in silage fermentation can utilize CA and CB_1_, such as sugars and starch, to produce lactic acid or ethanol to meet the needs for microbial growth during ensiling. As shown in Table 2, increased lactic acid and reduced WSC content among Inner Mongolian native grass silage strongly supported this result. Pires et al [26] examined the carbohydrate fraction in elephant grass silage and verified that the highest fraction was CB_2_, comprising more than 68.0% of CHO. In our study, CB_2_ also constituted the highest fraction of CHO, at 64.6%. The CB_2_ fraction is sensitive to the available NDF and CP content of grass silage, since grass silage provides the greatest amount of CB_2_ among all feeds in a simulated diet. Plants and microorganisms can produce some cellulolytic enzymes that dominate the process of soluble fiber degradation during ensiling, and some cellulase-like enzymes play a primary role in breaking down fiber. As CA, CB_1_, and CB_2_ decrease during ensiling, the carbohydrate fraction also decreases. The CC pool consists of unavailable cell wall carbohydrate including lignin. The NFC fraction includes macromolecular compounds; LAB and other microorganisms involved in silage fermentation cannot utilize CC. Therefore, CC and NFC content did not change significantly during ensiling.

### Changes in chemical composition during the aerobic stage

Aerobic stability is an important trait of silage; it determines the safety and quality of preserved forage upon exposure to air. Silage yeast can cause rapid aerobic deterioration of silage, leading to loss of silage nutrients in the aerobic stage [27]. As shown in Table 5, the chemical composition changed during aerobic exposure, including decreased amounts of OM, CP, and EE. It is possible that microbial activity during aerobic exposure degraded some of the CP and WSC. Lactate-assimilating yeasts metabolize WSC and fermentation end-products into carbon dioxide and water, leading to nutrient losses. Aerobic deterioration resulted in a higher amount of ADF, which is consistent with the findings of Chen and Weinberg [28]. This increase in ADF may have been due to the loss of soluble silage constituents during aerobic exposure, leaving higher concentrations of cell wall constituents. Cai et al [29] reported that the addition of *L. plantarum* to various types of forage improved silage fermentation but did not inhibit yeast growth or aerobic deterioration. In our study, the control and LAB-treated silages fermented well, but aerobic deterioration was not inhibited. Therefore, high-quality silage can contain large amounts of yeast and LAB [29], resulting in rapid spoilage of silage upon exposure to air.

### Changes in fermentation quality during the aerobic stage

Temperature and pH, as well as organic acid, yeast, and fungal content, are important indicators of aerobic stability in silage. The low lactic acid content and high pH observed in Inner Mongolian native grass silage during aerobic exposure in our study can be explained by the presence of lactic acid-utilizing epiphytic yeasts. Some yeasts convert lactic acid into carbon dioxide and ethanol under aerobic conditions, resulting in decreased lactic acid and increased pH [29]. A propionic acid-based preservative has been shown to markedly improve the aerobic stability of silage; however, no propionic or butyric acid was detected in the Inner Mongolian native grass silage examined in this study. It is possible that the high DM of the Inner Mongolian native grass silage limited the growth of *Clostridia* or propionic acid-producing bacteria. Ammonia-N content gradually increased during aerobic exposure in the Inner Mongolian native grass silage. This result may be due to the various microorganisms related to silage fermentation utilizing nitrogenous substances, such as protein and amino acids, and to decomposing N, leading to CP degradation and ammonia-N production during aerobic exposure.

### Changes in protein and carbohydrate fractions during the aerobic stage

Most yeast strains isolated from deteriorated silage have high lactic acid tolerance but low tolerance to propionic and butyric acids; therefore, they can grow at low pH and readily cause aerobic deterioration [29]. Aerobic bacteria, yeasts, and molds involved in silage fermentation can grow vigorously after the silo is opened, leading to aerobic deterioration. In this study, PA, PB_1_, PB_2_, PB_3_, and PC content decreased during aerobic exposure. Yeasts and molds are the major microorganisms causing aerobic deterioration; they utilize residual WSC, starch, and lactic acid to produce carbon dioxide and ethanol. The growth of aerobic bacteria, yeasts, or molds can influence PA, PB_1_, PB_2_, PB_3_, and PC fractions. CC and NFC content did not change significantly during aerobic exposure, likely because the microorganisms related to aerobic deterioration cannot use them for growth.

## CONCLUSION

In this study, we examined the dynamic changes in protein and carbohydrate fractions of Inner Mongolian native grass silage during ensiling and the aerobic stage in Inner Mongolia, China. The control and LAB-treated silages produced similar results in terms of fermentation quality, aerobic stability, and protein and carbohydrate fractions. Inner Mongolian native grass produced good silage, with nutrients being preserved during ensiling and protein and carbohydrate losses largely occurring during the aerobic stage.

## Figures and Tables

**Table 1:** Chemical composition of Inner Mongolian native grass silage during ensiling

Treatment	Ensiling (d)	OM	CP	EE	NDF	ADF	WSC

		-------------------------------------------------------------- % of DM ---------------------------------------------------------					
Control	0	93.38±0.65	10.76±0.19^a^	1.19±0.11	64.61±1.15	35.91±1.04	3.56±0.32^a^
	5	93.34±0.43	10.64±1.08^ab^	1.17±0.05	64.58±1.36	35.88±0.95	2.51±0.11^b^
	15	93.33±0.51	10.46±1.28^b^	1.16±0.26	64.53±1.03	35.86±0.98	1.17±1.05^c^
	20	93.31±0.54	10.44±0.98^b^	1.15±0.19	64.49±0.54	35.83±0.57	0.61±0.35^d^
	30	93.31±0.71	10.42±0.68^b^	1.13±0.19	64.46±1.06	35.82±0.55	0.49±0.19^d^
	60	93.30±0.72	10.41±0.55^b^	1.12±0.17	64.44±1.47	35.79±0.79	0.29±0.10^e^
LAB	0	93.38±0.65	10.76±0.49^a^	1.19±0.11	64.59±1.05	35.90±0.99	3.56±0.32^a^
	5	93.37±0.63	10.67±0.88^b^	1.18±0.05	64.56±1.01	35.86±1.10	2.22±0.62^b^
	15	93.35±0.85	10.47±0.95^c^	1.17±0.21	64.51±1.00	35.84±0.97	1.10±0.10^c^
	20	93.33±0.15	10.45±0.91^c^	1.13±0.16	64.46±0.56	35.82±0.45	0.82±0.24^d^
	30	93.32±0.72	10.44±0.61^c^	1.14±0.13	64.46±0.49	35.81±0.80	0.53±0.15^e^
	60	93.31±0.76	10.42±0.74^c^	1.13±0.19	64.42±1.02	35.74±0.51	0.31±0.07^f^
SEM		0.37	0.56	0.03	0.59	0.04	0.05
Significance of main effects and interaction							
Additives (A)		0.9297	0.6529	0.9355	0.9587	0.9435	0.5179
Ensiling d (D)		0.9999	<0.0001	0.9775	0.9997	0.9998	<0.0001
A×D		1.0000	0.0010	0.9998	0.9999	0.9998	0.0008

Silage data are the average of three silage samples.

OM, organic matter; CP, crude protein; EE, ether extract; NDF, neutral detergent fiber; ADF, acid detergent fiber; WSC, water-soluble carbohydrates; DM, dry matter; LAB, lactic acid bacteria inoculant Chikuso-1; SEM, standard error of the mean.

a–f Means with different superscripts within a column are significantly different (p≤0.05).

**Table 2:** Fermentation quality of Inner Mongolian native grass silage during ensiling

Treatment	Ensiling (d)	DM (%)	pH	Lactic acid	Acetic acid	Propionic acid	Butyric acid	Ammonia-N g/kg of FM

				----------------------------------- % of FM -----------------------------				
Control	0	54.92±0.70	6.48±0.74^a^	ND	ND	ND	ND	ND
	5	54.58±0.14	4.55±1.16^b^	0.57±0.18^b^	0.40±0.15^b^	ND	ND	0.39±0.21^d^
	15	53.96±1.44	4.36±1.01^bc^	0.92±0.16^a^	0.41±0.24^a^	ND	ND	0.41±0.11^c^
	20	53.54±1.19	4.30±0.98^c^	0.95±0.09^a^	0.42±0.16^a^	ND	ND	0.43±0.16^b^
	30	53.51±0.97	4.20±0.70^c^	1.00±0.19^a^	0.44±0.09^a^	ND	ND	0.44±0.13^b^
	60	53.50±1.01	4.19±0.83^c^	1.03±0.23^a^	0.45±0.19^a^	ND	ND	0.47±0.21^a^
LAB	0	56.92±0.70	6.48±0.74^a^	ND	ND	ND	ND	ND
	5	54.47±1.27	4.54±0.84^b^	0.62±0.27^b^	0.44±0.18^b^	ND	ND	0.38±0.22^c^
	15	53.90±0.99	4.30±0.95^c^	0.94±0.39^ab^	0.46±0.23^a^	ND	ND	0.41±0.21^b^
	20	53.49±2.01	4.22±0.59^c^	0.99±0.14^a^	0.45±0.13^a^	ND	ND	0.42±0.39^b^
	30	53.48±1.17	4.19±0.20^c^	1.05±0.35^a^	0.42±0.09^a^	ND	ND	0.44±0.32^a^
	60	53.46±0.98	4.17±0.76^c^	1.09±0.15^a^	0.42±0.16^a^	ND	ND	0.46±0.31^a^
SEM		1.21	0.27	0.18	0.13	-	-	0.22
Significance of main effects and interaction								
Additives (A)		0.6813	0.4550	0.3916	0.5066	-	-	0.4312
Ensiling d (D)		0.9234	<0.0001	<0.0001	<0.0001	-	-	<0.0001
A×D		0.9999	0.9862	0.9993	0.6564	--	-	-

Silage data are the average of three silage samples.

DM, dry matter; FM, fresh matter; LAB, lactic acid bacteria Chikuso-1 inoculant; SEM, standard error of the mean, data are the average of three silage samples; ND, not detected as below 0.01% of FM; “-” means the value is zero.

a–d Means with different superscripts within a column are significantly different (p≤0.05).

**Table 3:** The CNCPS protein fractions of Inner Mongolian native grass and silage during ensiling

Treatment	Ensiling (d)	PA1)	PB_1_1)	PB_2_1)	PB_3_1)	PC1)

		----------------------------------------------------- % f CP ----------------------------------------------------------------------------				
Control	0	35.62±1.32^b^	9.33±1.14^a^	42.38±1.24^a^	10.84±0.94	2.83±1.20
	5	37.77±2.17^a^	8.08±2.60^b^	40.89±1.20^b^	10.67±0.98	2.58±1.79
	15	37.84±1.49^a^	7.97±1.69^b^	40.79±1.59^b^	10.62±0.98	2.77±1.80
	20	37.93±1.05^a^	7.90±0.90^b^	40.70±2.66^b^	10.60±0.36	2.87±0.61
	30	37.97±1.11^a^	7.80±0.89^b^	40.49±1.04^b^	10.57±0.94	3.18±0.99
	60	38.07±0.93^a^	7.78±0.99^b^	40.37±1.36^b^	10.51±1.16	3.27±1.28
LAB	0	35.62±1.32^b^	9.33±1.14^a^	42.38±1.24^a^	10.84±0.94	2.83±1.20
	5	37.65±1.04^a^	8.11±0.90^b^	40.94±1.23^b^	10.65±1.16	2.65±1.54
	15	37.82±0.94^a^	8.04±1.63^b^	40.84±2.47^b^	10.61±1.46	2.68±1.34
	20	37.92±1.12^a^	7.93±1.11^b^	40.73±0.63^b^	10.58±1.65	2.83±1.32
	30	37.95±2.05^a^	7.83±2.10^b^	40.51±1.28^b^	10.54±1.06	3.16±1.39
	60	38.00±1.50^a^	7.79±1.08^b^	40.39±1.36^b^	10.47±1.04	3.36±0.86
SEM		1.14	1.22	1.23	1.16	0.50
Significance of main effects and interaction						
Additives (A)		0.4852	0.8829	0.8218	0.8223	0.9970
Ensiling d (D)		<0.0001	0.0003	<0.0001	0.3514	0.7163
A×D		0.9975	1.0000	0.9999	1.0000	1.0000

Silage data are the average of three silage samples.

CNCPS, Cornell Net Carbohydrate and Protein System; CP, crude protein; LAB, lactic acid bacteria inoculant Chikuso-1; SEM, standard error of the mean.

1) PA, non-protein nitrogen; PB_1_, true protein degraded rapidly; PB_2_, true protein degraded intermediately; PB_3_, true protein degraded slowly; PC, bound true protein.

a,b Means with different superscripts within a column are significantly different (p≤0.05).

**Table 4:** The CNCPS carbohydrate fractions of Inner Mongolian native grass silage during ensiling

Treatment	Ensiling d	CHO (% of DM)	Carbohydrate fraction (% of CHO)1)				NFC % of CHO

			CA	CB_1_	CB_2_	CC	
Control	0	84.29±2.10^a^	12.93±2.06^a^	2.34±0.83^a^	64.57±1.16^a^	4.45±0.63	31.22±1.19
	5	78.43±5.66^b^	7.31±1.04^b^	2.31±1.04^a^	64.36±3.32^ab^	4.46±0.71	31.18±0.64
	15	78.21±4.10^b^	7.21±0.95^c^	2.26±1.05^a^	64.28±3.05^b^	4.47±1.01	31.25±0.95
	20	78.09±4.45^b^	7.15±1.03^cd^	2.24±0.97^a^	64.22±2.05^b^	4.48±1.02	31.30±0.48
	30	78.03±3.01^b^	7.14±2.03^cd^	2.22±0.62^a^	64.19±1.01^b^	4.49±0.97	31.32±1.06
	60	77.97±3.62^b^	7.13±1.82^d^	2.19±0.70^a^	64.18±2.06^b^	4.48±0.67	31.35±0.63
LAB	0	84.29±2.10^a^	12.93±2.06^a^	2.34±0.83^a^	64.57±1.16^a^	4.45±0.63	31.22±1.19
	5	78.45±5.25^b^	7.33±3.03^b^	2.33±1.03^ab^	64.32±3.04^a^	4.46±0.62	31.23±0.80
	15	78.23±3.16^b^	7.24±1.13^bc^	2.27±1.08abc	64.26±3.16^a^	4.47±1.02	31.27±0.02
	20	78.11±4.12^b^	7.18±1.12^c^	2.25±0.96^bc^	64.20±2.01^a^	4.49±1.02	31.32±0.65
	30	78.04±3.70^b^	7.15±2.02^c^	2.24±0.06^bc^	64.17±1.54^a^	4.48±0.88	31.35±1.11
	60	77.98±3.18^b^	7.14±1.02^c^	2.21±0.75^c^	64.15±2.10^a^	4.49±0.72	31.36±2.20
SEM		3.37	1.04	1.04	2.11	1.02	3.32
Significance of main effects and interaction							
Additives (A)		0.8730	0.4358	0.5371	0.7627	0.8360	0.8074
Ensiling d (D)		<0.0001	<0.0001	0.0150	0.0198	0.5819	0.9868
A×D		0.9997	0.9957	0.9999	0.9997	0.9977	0.9999

Silage data are the average of three silage samples.

CNCPS, Cornell Net Carbohydrate and Protein System; DM, dry matter; CHO, total carbohydrate; NFC, non-fibrous carbohydrate; LAB, lactic acid inoculant Chikuso-1; SEM, standard error of the mean.

1) CA, sugars; CB_1_, starch; CB_2_, soluble fiber; CC, undegradable neutral detergent fiber.

a–d Means with different superscripts within a column are significantly different (p≤0.05).

**Table 5:** Chemical composition of Inner Mongolian native grass silage during aerobic stage

Treatment	Stage (d)	OM	CP	EE	NDF	ADF	WSC

		------------------------------------------------------------ % of DM -----------------------------------------------------------------					
Control	0.5	93.15±2.12^a^	10.40±2.39^a^	1.15±0.89^a^	65.99±2.96^a^	33.10±3.70^c^	0.26±0.11^a^
	2	92.18±3.13^b^	10.18±1.04^a^	1.12±0.90^a^	65.12±3.26^a^	33.33±3.28^c^	0.20±0.17^b^
	5	91.85±5.15^bc^	7.61±2.22^b^	1.06±0.41^ab^	63.34±5.07^b^	35.77±2.37^b^	0.12±0.15^c^
	10	91.63±3.35^c^	7.47±1.22^b^	0.90±0.82^b^	63.13±4.19^b^	36.59±4.16^a^	0.08±0.09^d^
LAB	0.5	93.26±3.59^a^	10.41±2.26^a^	1.17±0.87^a^	65.86±2.11^a^	33.02±4.33^c^	0.28±0.13^a^
	2	92.38±3.10^b^	10.20±1.13^a^	1.14±0.92^a^	64.95±2.07^b^	33.28±3.09^c^	0.21±0.12^b^
	5	91.94±4.07^b^	7.65±3.22^b^	1.09±0.57^a^	63.30±3.25^c^	35.73±3.58^b^	0.13±0.13^c^
	10	91.86±3.09^b^	7.55±2.11^b^	0.92±0.94^b^	63.09±4.27^c^	36.53±4.31^a^	0.10±0.12^c^
SEM		3.15	2.13	0.74	2.22	3.23	0.21
Significance of main effects and interaction							
Additives (A)		0.1611	0.6602	0.4044	0.5527	0.7219	0.0987
Stage d (D)		<0.0001	<0.0001	<0.0001	<0.0001	<0.0001	<0.0001
A×D		0.9623	0.9944	0.9981	0.9868	0.9868	0.9998

Aerobic stage data are the average of three aerobic samples.

OM, organic matter; CP, crude protein; EE, ether extract; NDF, neutral detergent fiber; ADF, acid detergent fiber; WSC, water-soluble carbohydrates; DM, dry matter; LAB, Lactic acid bacteria inoculant Chikuso-1; SEM, standard error of the mean.

a–d Means with different superscripts within a column are significantly different (p≤0.05).

**Table 6:** Changes in fermentation quality of Inner Mongolian native grass silage during aerobic stage

Treatment	Stage (d)	DM (%)	pH	Lactic acid	Acetic acid	Propionic acid	Butyric acid	Ammonia-N (g/kg of FM)

				----------------------------------- % of FM-----------------------------------				
Control	0.5	53.47±1.27^c^	4.25±1.20^c^	1.01±0.46^a^	0.43±0.25^a^	ND	ND	0.57±0.12^d^
	2	55.69±1.24^b^	4.77±0.93^b^	0.88±0.12^b^	0.40±0.14^a^	ND	ND	0.67±0.31^c^
	5	56.08±0.90^ab^	4.85±1.09^b^	0.69±0.21^c^	0.36±0.23^a^	ND	ND	0.74±0.12^b^
	10	56.32±3.22^a^	5.18±2.09^a^	0.36±0.19^d^	0.23±0.11^b^	ND	ND	0.82±0.31^a^
LAB	0.5	53.44±1.33^c^	4.18±1.07^c^	1.05±0.46^a^	0.44±0.26^a^	ND	ND	0.56±0.11^d^
	2	55.57±1.06^b^	4.67±0.99^b^	0.89±0.23^b^	0.41±0.14^a^	ND	ND	0.66±0.22^c^
	5	56.04±1.34^ab^	4.81±1.10^b^	0.71±0.23^c^	0.38±0.24^a^	ND	ND	0.73±0.11^b^
	10	56.28±2.33^a^	5.15±1.05^a^	0.38±0.25^d^	0.24±0.14^b^	ND	ND	0.81±0.32^a^
SEM		1.25	1.26	0.02	0.22	-	-	0.22
Significance of main effects and interaction								
Additives (A)		0.6076	0.2071	0.0864	0.4814	-	-	0.1892
Stage d (D)		<0.0001	<0.0001	<0.0001	<0.0001	-	-	<0.0001
A×D		0.9884	0.9338	0.8471	0.9975	-	-	0.8530

Aerobic stage data are the average of three aerobic samples.

DM, dry matter; FM, fresh matter; ND, not detected as below 0.01% of FM; LAB, lactic acid bacteria inoculant Chikuso-1; SEM, standard error of the mean; “-” means the value is zero.

a–d Means with different superscripts within a column are significantly different (p≤0.05).

**Table 7:** Changes in the CNCPS protein fractions of Inner Mongolian native grass silage during aerobic stage

Treatment	Stage (d)	PA1)	PB_1_1)	PB_2_1)	PB_3_1)	PC1)

		------------------------------------------------------- % of CP --------------------------------------------------------------------------				
Control	0.5	38.09±2.13^b^	7.20±3.06^a^	40.36±3.37^a^	9.83±1.21^a^	4.52±2.36^c^
	2	38.27±3.08^b^	7.12±2.07^a^	39.73±2.18^b^	9.72±2.18^a^	5.16±1.15^c^
	5	39.39±3.21^a^	6.52±3.06^b^	37.68±2.26^c^	8.83±5.38^b^	7.58±2.56^b^
	10	39.43±2.02^a^	6.39±2.02^c^	37.52±1.30^c^	8.34±4.37^b^	8.31±2.19^a^
LAB	0.5	38.06±2.05^b^	7.21±3.04^a^	40.38±3.15^a^	9.86±2.19^a^	4.49±1.22^d^
	2	38.26±3.03^b^	7.14±2.04^b^	39.77±2.18^b^	9.74±2.07^a^	5.09±2.11^c^
	5	39.35±3.35^a^	6.56±2.03^c^	37.72±2.04^c^	8.85±3.28^b^	7.52±2.29^b^
	10	39.41±2.01^a^	6.40±2.02^d^	37.53±1.05^c^	8.38±4.13^c^	8.29±1.12^a^
SEM		3.09	2.03	1.13	3.14	2.35
Significance of main effects and interaction						
Additives (A)		0.7503	0.3834	0.7493	0.8142	0.4625
Stage d (D)		<0.0001	<0.0001	<0.0001	<0.0001	<0.0001
A×D		0.9985	0.9351	0.9986	0.9998	0.7930

Aerobic stage data are the average of three aerobic samples.

CNCPS, Cornell Net Carbohydrate and Protein System; CP, crude protein; LAB, lactic acid bacteria inoculant Chikuso-1; SEM, standard error of the mean.

1) PA, non-protein nitrogen; PB_1_, true protein degraded rapidly; PB_2_, true protein degraded intermediately; PB_3_, true protein degraded slowly; PC, bound true protein.

a–d Means with different superscripts within a column are significantly different (p≤0.05).

**Table 8:** The CNCPS carbohydrate fractions of Inner Mongolian native grass silage during aerobic stage

Treatment	Stage (d)	CHO (% of DM)	Carbohydrate fraction (% of CHO)1)				NFC (% of CHO)

			CA	CB_1_	CB_2_	CC	
Control	0.5	77.71±2.43^a^	7.10±1.08^a^	2.18±1.05^a^	64.16±3.07^a^	4.27±1.09	31.57±1.54
	2	76.56±1.04^b^	6.94±1.17^a^	1.56±1.06^b^	63.84±4.03^ab^	4.22±2.41	31.94±2.36
	5	72.36±2.34^c^	3.37±2.08^b^	1.16±2.06^c^	63.40±2.22^bc^	4.43±1.14	32.17±1.19
	10	71.68±3.15^c^	3.31±1.43^b^	1.15±1.01^c^	63.12±3.60^c^	4.10±1.41	32.78±1.76
LAB	0.5	77.75±2.45^a^	7.11±2.01^a^	2.20±1.05^a^	64.15±2.01^a^	4.29±2.21	31.56±1.66
	2	76.43±1.07^b^	6.98±1.14^a^	1.57±2.01^b^	63.75±3.85^a^	4.13±1.34	32.12±2.67
	5	72.28±3.32^c^	3.43±1.33^b^	1.17±1.01^c^	63.39±4.54^a^	4.29±1.06	32.32±1.57
	10	71.89±2.14^c^	3.39±0.99^b^	1.16±1.03^c^	63.07±2.43^a^	4.27±1.18	32.66±1.51
SEM		2.17	2.31	1.02	2.26	1.27	1.32
Significance of main effects and interaction							
Additives (A)		0.9717	0.6348	0.3810	0.8308	0.9721	0.8302
Stage d (D)		<0.0001	<0.0001	<0.0001	0.0051	0.8844	0.5181
A×D		0.9632	0.9949	0.9888	0.9982	0.9378	0.9605

Aerobic stage data are the average of three aerobic samples.

CNCPS, Cornell Net Carbohydrate and Protein System; CHO, total carbohydrate; DM, dry matter; NFC, non-fibrous carbohydrate; LAB, lactic acid inoculant Chikuso-1; SEM, standard error of the mean.

1) CA, sugars; CB_1_, starch; CB_2_, soluble fiber; CC, undegradable neutral detergent fiber.

a–c Means with different superscripts within a column are significantly different (p≤0.05).
